# Supplementary material for: The Effectiveness of the Low-Glycemic and Insulinemic (LOGI) Regimen in Maintaining the Benefits of the VLCKD in Fibromyalgia Patients
Source: Nutrients. 2024 Nov 30;16(23):4161. doi: 10.3390/nu16234161 (PMC11643857; doi:10.3390/nu16234161)
Supplement: Supplementary file 1 [file nutrients-16-04161-s001.zip › nutrients-3318167-supplementary.pdf]

Table S1. Clinical parameters related to serum and urine of fibromyalgia women calculated before (t0) and after 45 (t45) and 90 (t90) days of diet. The table shows the average value (Mean) and the relative standard deviation (Dev.std),p.value calculated using T-test comparing t0 vs t45 and t0 vs t90.

		First Group			Second group			
**Parameters**		**Mean**	**Dev.std**	**p-value**	**Mean**	**Dev.std**	**p-value**	**Reference value**
	t0	32,29	8,04		33,85	6,57		mg/dL 10-50
	t45	35,29	7,90	NS	27,77	7,32	NS	
	t90	34,19	9,33	NS	34,15	9,39	NS	
Glucose	t0	82,90	4,67		86,46	4,25		mg/dL 70-105
	t45	81,90	9,38	NS	88,23	7,54	NS	
	t90	82,05	6,77	0,0361	85,69	6,05	NS	
Creatinine	t0	0,70	0,09		0,70	0,09		mg/dL0,57-1,11
	t45	0,72	0,08	0,0005	0,72	0,12	NS	
	t90	0,73	0,12	0,0224	0,68	0,07	NS	
AST	t0	18,90	6,92		19,00	7,84		IU/L 5-34
	t45	19,81	5,22	0,4568	19,08	5,78	NS	
	t90	16,43	3,90	0,0027	21,23	9,15	NS	
ALT	t0	19,95	14,23		23,92	15,79		IU/L 0-55
	t45	22,10	10,07	0,0114	22,08	12,40	NS	
	t90	18,00	7,39	0,0082	23,23	15,08	NS	
GGT	t0	25,00	40,98		20,85	11,99		IU/L 9-36
	t45	15,43	16,13	NS	17,08	9,69	NS	
	t90	23,00	36,69	NS	14,00	3,49	NS	
Col tot	t0	206,60	32,76		208,85	32,73		mg/dL ≤200
	t45	191,50	44,97	0,0046	174,46	28,04	NS	
	t90	200,20	43,28	NS	185,08	27,90	NS	
HDL	t0	66,62	15,98		55,85	11,43		mg/dL Optimal: superiore a 65
	t45	51,33	13,36	6,69E-07	46,85	8,46	0,03	Moderate risk:45-65
	t90	54,71	13,95	0,0144	50,69	7,90	NS	Hight risk: ≤ a 45
LDL	t0	131,50	32,22		139,23	23,67		mg/dL Optimal: fino a 130
	t45	123,90	44,05	0,0026	112,54	26,57	0,01	Moderate Risk :130-159
	t90	127,40	38,87	0,1484	113,31	19,70	0,01	Hight Risk: superiore a 160
Tryglicerid	t0	109,80	73,33		92,00	32,30		mg/dL ≤150
	t45	80,43	31,40	0,00004	65,62	18,72	0,02	
	t90	91,86	54,31	0,0056	67,54	22,82	0,04	
Transferrine	t0	329,00	58,94		291,15	27,52		mg/dL 180-382
	t45	295,50	70,19	8,44E-06	264,62	32,20	0,03	
	t90	306,00	56,66	0,0051	259,08	37,33	0,02	
Folic Acid	t0	6,17	3,20		7,05	4,15		ng/mL 2,80-12,40
	t45	7,70	2,66	7,08E-05	9,40	4,49	NS	
	t90	6,71	3,03	NS	8,76	4,12	NS	
Vitamin B12	t0	401,90	132,91		552,58	274,19		pg/mL

	t45	461,00	197,04	3,30E-05	656,46	425,68	NS	187-883
	t90	416,10	194,87	NS	612,31	345,94	NS	
Insulinemia	t0	7,34	3,36		8,38	7,67		mcUI/mL<br>0,0-25,0
	t45	5,10	2,65	0,0186	5,96	3,51	NS	
	t90	6,08	2,43	NS	5,64	5,50	NS	
Cortisol	t0	8,64	3,39		5,81	1,54		µg/dL<br>3,7-19,4
	t45	8,69	3,41	0,0411	6,92	2,68	NS	
	t90	7,80	2,92	NS	7,91	2,79	NS	
Fibrinogen	t0	327,30	76,38		355,00	81,29		mg/dL 180-350
	t45	365,00	61,46	0,0084	379,08	80,77	NS	
	t90	357,00	68,78	NS	339,46	80,56	NS	
HB	t0	12,60	0,78		13,57	1,44		g/dL<br>13,0-17,0
	t45	12,89	0,88	3,31E-06	13,46	1,24	NS	
	t90	13,13	1,03	NS	13,48	1,46	NS	
HCT	t0	36,83	2,20		40,29	4,21		%<br>38,0-49,0
	t45	37,52	2,60	0,00013	38,99	2,69	NS	
	t90	38,86	3,19	NS	40,41	3,59	NS	
Urine pH	t0	5,76	0,51		5,62	0,30		pH<br>5,0-6,5
	t45	3,53	0,79	NS	5,88	0,74	NS	
	t90	6,02	0,91	NS	5,83	0,69	NS	
HOMA	t0	1,51	0,61		1,37	0,31		mg/dL 0,23 – 2,5
	t45	1,06	0,64	0,032	1,07	0,66	0,03	
	t90	1,23	0,30	NS	1,21	0,23	NS	
uric acid	t0	4,30	1,59		4,44	0,70		mg/dL 2,6-6,0
	t45	4,58	1,50	9,29E-05	4,72	1,08	NS	
	t90	4,27	1,40	0,0093	4,60	0,89	NS	
HTSH	t0	1,30	0,70		1,064	0,69		µIU/mL <6 months :0,3700-5,5400<br>6mm-14aa:0,6100-4,4300<br>15-19anni:0,2500-3,4500<br>Adults :0,3500-4,9400
	t45	1,23	0,76	0,017	0,966	0,62	NS	
	t90	1,18	0,67	NS	1,210	0,87	NS	
FT3	t0	2,81	0,44		2,838	0,87		pg/mL<br><1anno:2,23-5,19<br>1-12anni:2,74-4,49<br>13-15anni:2,43-4,02<br>16-19anni:2,01-3,59<br>Adults1,71-3,71
	t45	2,47	0,31	NS	2,687	0,32	NS	
	t90	2,56	0,55	NS	2,543	0,26	0,03	
FT4	t0	0,95	0,10		0,995	1,71		ng/dL<br>5-14giorni:0,87-3,35<br>15-29giorni:0,55-2,65<br>30gg-1anno:0,83-1,84<br>2-19anni:0,86-1,39<br>Adults:0,70-1,48
	t45	1,01	0,14	0,0069	1,057	0,27	NS	
	t90	1,04	0,21	NS	1,112	0,00	0,08	
C protein	t0	0,61	0,61		0,36	1,49		mg/dL ≤0,50
	t45	0,40	1,06		0,25	0,45	NS	

	t90	0,37	0,41		0,26	0,04	NS	
25 OHD	t0	28,90	1,26		35,77	1,27		ng/mL<br>DEFICIENCIES:fino a 10<br>INSUFFICIENCY:11-30<br>SUFFICIENCY:31-100<br>TOXICITY':>100
	t45	42,57	1,27	4,12E-02	34,46	0,52	NS	
	t90	42,52	1,51	0,015	33,08	0,04	NS	
testosterone	t0	30,81	9,02		26,92	11,59		ng/dL<br><15(7-9anni)<br>2-42(10-11anni)<br>6-64(12-13anni)<br>9-49(14-15anni)<br>8-63(16-17anni)<br>10-59(18-50anni)<br>6-25(>51anni)
	t45	29,67	11,70	1,18E-05	31,31	138,41	NS	
	t90	24,00	7,50	0,0020	28,08	149,58	NS	
estradiol	t0	92,95	94,34		112,83	149,58		pg/mL<br>21-251 follicolar<br>38-649 pick<br>21-312 luteinic<br>10-28 postmaenopause
	t45	116,00	130,79	NS	107,62	63,27	NS	
	t90	94,24	83,67	NS	121,00	47,45	NS	
SHBG	t0	146,50	160,74		81,31	4,15		nmol/L<br>18-114
	t45	175,80	172,68	6,44E-12	93,85	4,49	NS	
	t90	146,30	193,66	NS	107,08	4,12	NS	

ALT: Alanine aminotransferase; AST: Aspartate amminotransferase; GGT: Gamma glutamil transferase ;HDL: High density lipoprotein ;LDL: Low density lipoprotein; HTSH: Thyroid-Stimulating Hormone; FT3: tri-iodothyronine; FT4: thyroxine;25 OHD: calcidiol; SHGB: Sex hormone binding globulin; HB: hemoglobin; HCT: Hematocrit; HOMA: Homeostasis Model Assessment

Table S2. PLS-DA classification of the five components (comps) based on accuracy, R2, Q2 related to fibromyalgia patients' serum, urine and salivary extract before and after nutritional regime, carried out by NMR.

**MEASURE SERUM**	**1 COMP**	**2 COMP**	**3COMP**	**4COMP**	**5COMP**
**Accurancy**	0.60	0.77	0.94	0.94	0.93
**Q2**	0.72	0.95	0.95	0.96	0.96
**R2**	0.70	0.93	0.92	0.92	0.91
**MEASURE URINE**	**1 COMP**	**2 COMP**	**3COMP**	**4COMP**	**5COMP**
**Accurancy**	0.73	0.76	0.84	0.90	1.0
**Q2**	0.88	0.91	0.98	0.98	0.99
**R2**	0.86	0.91	0.97	0.98	0.98
